# Supplementary material for: Coverage of suicide deaths in newspapers and perceptions of suicide loss survivors on reporting: Insights from a community survey in India
Source: PLOS Ment Health. 2025 Oct 28;2(10):e0000473. doi: 10.1371/journal.pmen.0000473 (PMC12798486; doi:10.1371/journal.pmen.0000473)
Supplement: Attachment — Submitted filename: 251008 Response to reviewer comments.docx [file pmen.0000473.s002.docx]

8 October 2025

Dear Editor,

Thank you for inviting revision of our manuscript. We are grateful to the reviewers for the detailed and constructive comments. We believe that the manuscript has improved significantly by these comments. We have responded to the points raised by the reviewers in detail as documented below. The line numbers indicated in the response refer to those in the clean revised version of the manuscript.

**Journal Requirements**

1. Please provide a detailed online Financial Disclosure statement. This is published with the article. It must therefore be completed in full sentences and contain the exact wording you wish to be published.
a) State the initials, alongside each funding source, of each author to receive each grant, if applicable. For example: “This work was supported by the National Institutes of Health (####### to AM; ###### to CJ) and the National Science Foundation (###### to AM).”

**Thank you. We have now updated the statement as required.**

b) State what role the funders took in the study. If the funders had no role in your study, please state: “The funders had no role in study design, data collection and analysis, decision to publish, or preparation of the manuscript.”

**Thank you. We have now updated the statement.**

2. Please ensure that the funders and grant numbers match between the Financial Disclosure field and the Funding Information tab in your submission form. Note that the funders must be provided in the same order in both places as well.

**Thank you, noted.**

3. Please update your online Competing Interests statement. If you have no competing interests to declare, please state: “The authors have declared that no competing interests exist”.

**Thank you. We have now updated the statement.**

4. If the reviewer comments include a recommendation to cite specific previously published works, please review and evaluate these publications to determine whether they are relevant and should be cited. There is no requirement to cite these works unless the editor has indicated otherwise.

**Thank you, noted.**

**Additional Editor Comment**

Please remove all causal languages, including increase, affect, influence, and replace them with associated with, related to, or covary with to reflect your cross-sectional design. Thank you!

**Thank you. We have made replacements in lines 42, 235-236, 241, 288 293 and 352.**

**Reviewer #1**

General Comments:
This manuscript presents a timely and culturally relevant study on newspaper coverage of suicide deaths and the perceptions of suicide loss survivors in India. The topic is understudied, especially in low- and middle-income countries, and the findings have significant implications for media guidelines, postvention support, and public health policy. The methodology is generally sound, and the mixed-methods approach adds depth to the quantitative findings. However, several aspects require clarification and strengthening to enhance the manuscript's rigor and impact.

**Thank you, we have revised the manuscript.**

Major Comments:

1. Clarity on Sampling and Representativeness: The manuscript states that 155 suicide deaths were included from three states, but it is unclear how these were selected from the larger national survey of 366 suicide deaths. Please clarify the sampling strategy and justify the representativeness of the selected states and cases (e.g., are there any notable socioeconomic, cultural, or other differences between the three states selected in the article and other states).

**We have added extensive details on the national survey, which was designed to capture a representative sample of deaths between January 2019 and December 2022 in India (lines 104-126. The three states for this study were selected as these accounted for 60% of all suicide deaths in the national survey, and represented varied socio-demography in India, with Maharashtra and Tamil Nadu reflect stronger health systems relative to Uttar Pradesh (lines 128-130.**

2. Definition and Recruitment of “Suicide Loss Survivors”: The term “adult member most knowledgeable about the suicide” is used to define survivors. Please provide more detail on how this person was identified and whether this introduced any selection bias (e.g., gender, relationship to deceased).

**All members of the household of the deceased are suicide loss survivors. The household decided who was best placed to respond to the study tools given the variety that was covered in the interview. In India, typically, the head of the household provide such interviews, who are mostly men.**

3. Qualitative Analysis Methods: The inductive thematic analysis process is briefly mentioned but not sufficiently detailed. Please describe the coding process, how themes were derived, inter-coder reliability, and how disagreements were resolved.

**We have added more details in lines 140-149**

4. Missing Demographic and Contextual Data: The socio-demographic table for survivors (Table 2) is incomplete and difficult to interpret. Please ensure all rows and columns are clearly labeled and that p-values are correctly associated with the relevant comparisons.

**We re-checked Table 2. It is complete with no missing values, and has all rows and columns clearly labelled with p-values.**

5. Theoretical Framework: The study would benefit from a stronger theoretical grounding in grief, trauma, or stigma theories to frame the findings and discussion. For instance, incorporating models of complicated grief or structural stigma could deepen the interpretation of survivors’ responses.

**Given the limited prior research on how families experience media reports of suicide deaths, the study was intentionally exploratory. Anchoring the work in grief, trauma, or stigma frameworks could have risked narrowing the inquiry and pre-shaping participant accounts. Instead, an open, descriptive approach was chosen to allow unanticipated themes to emerge. The findings provide a foundation upon which future research can more critically engage with existing theoretical frameworks or develop new ones. We have now indicated this in discussion lines 303-310.**

6. Implications for Practice: While the discussion touches on media guidelines, more specific recommendations for journalists, policymakers, and mental health professionals in the Indian context would strengthen the practical impact of the study.

**We have attempted more specificity in our recommendations in lines 358-377.**

Minor Comments:

7. Abstract: The abstract clearly summarizes key findings but could briefly mention the methodological approach (semi-qualitative interviews) and the three states involved.

**We have added three states in line 34-35 We have replaced semi-qualitative with semi-structured interviews in line 33**

8. Introduction: The introduction effectively sets the context but could better highlight the gap regarding survivors’ perspectives, which is the study’s main contribution.

**We have now improved the context in lines 70-78.**

9. Results: Some confidence intervals are very wide due to small sample sizes (e.g., female suicides in certain categories). Acknowledge this limitation when interpreting these estimates.

**This is now added in lines 280-281in discussion.**

10. Tables and Figures: Figures 1 and 2 are too blurry to see their contents. High-definition images are recommended.

**We have updated the Figures to improve the clarity.**

11. Language and Flow: The manuscript is well-written but would benefit from minor editing for flow and consistency (e.g., avoid repetition in the discussion).

**We have attempted to address this issue in the revised manuscript.**

Conclusion:
This study addresses an important gap in the literature on suicide reporting and its impact on survivors in India. With revisions to clarify methods, strengthen the theoretical framework, and expand practical implications, this manuscript will be a valuable contribution to the field of mental health and media studies.

**Thank you.**

**Reviewer #2**

Thank you for submitting your manuscript entitled “Coverage of suicide deaths in newspapers and perceptions of suicide loss survivors on reporting: Insights from a community survey in India” to PLOS Mental Health. I found the paper to be timely, original, and of potential high value to the field. In particular, the inclusion of survivor perspectives adds important depth to an under-researched area. However, before this manuscript can be considered further, several substantive revisions are needed.

**Thank you, we have revised the manuscript.**

**Major points:**

1. Please strengthen the **conceptual framing** in the introduction and discussion. Situate survivor perceptions more explicitly within theories of bereavement, stigma, and suicide postvention.

**Given the limited prior research on how families experience media reports of suicide deaths, the study was intentionally exploratory. Anchoring the work in grief, trauma, or stigma frameworks could have risked narrowing the inquiry and pre-shaping participant accounts. Instead, an open, descriptive approach was chosen to allow unanticipated themes to emerge. The findings provide a foundation upon which future research can more critically engage with existing theoretical frameworks or develop new ones.**

2. Provide greater detail and transparency in the **methods**, particularly around survivor selection, ethical safeguards for bereaved participants, and the qualitative analysis process.

**We have added more details on the ethical safeguards (91-99), survivor selection (lines 133-135), and the qualitative analysis process (lines 140-149).**

3. Consider restructuring the **results presentation** to make tables and figures more concise and interpretable. Highlight statistically significant results clearly while avoiding overinterpretation of non-significant trends.

**We have reviewed the Tables and Figures again to assess interpretability. These seem ok to us. We have deleted some sections in the results to avoid overinterpretation of non-significant trends.**

4. Enhance the **discussion** by systematically integrating survivor narratives, acknowledging study limitations more explicitly, and contextualizing findings within both Indian and international suicide reporting guidelines.

**We have strengthened the discussion by expanding on the potential role of the study findings in suicide reporting guidelines (lines 366-377). Limitations are now explicitly addressed in lines 303-315. Also, we have now numbered the cases in Table 3, and have indicated this numbering in the discussion to integrate the survivor narratives (lines 318-347) and also have provided examples from our cases (lines340-345).**

5. Revise the **abstract** for clarity and alignment with journal requirements (clearer objective, concise methods, and implications).

**We have updated the abstracts (lines 33-35and 50-52 to improve clarity.**

**Minor points:**

6. Address typographical inconsistencies.

**We have checked the manuscript.**

7. Refine reference list for relevance.

**We have added more references.**

8. Clarify terminology (e.g., “semi-qualitative interviews”).

**Thank you. We have replaced semi-qualitative interviews with semi-structured interviews, as the latter reflects the methods more appropriately. Interview was undertaken by trained interviewers as mentioned in lines 130-142.**

Overall, I believe this paper has the potential to make a meaningful contribution, but it requires major revisions to improve clarity, rigor, and international relevance. I look forward to reviewing a revised version.

**Thank you.**

**Reviewer #3**

The article was well researched and the findings speak to issues that need to be worked on. If these findings are looked into and reviewed there could be lobbying for change in some reporting policies. As always the elite tend to be all over the media hence the high coverage of their suicide cases yet the marginalized cases are key to reform in some policies. I noted cultural beliefs, social status also played a role in reporting suicide cases which indicates a lot was done to obtain the data.

**Thank you very much for this appreciation.**

**Reviewer #4**

General comments:
The paper is addressing the aspect of coverage of suicide deaths in newspapers and perceptions of suicide loss survivors on reporting following the community survey in India. However, there issues that needs to be addressed.

**Thank you, we have revised the manuscript.**

1. The authors need to define some terms such as “Suicide loss survivors” as used in the manuscript. There is also some minor grammar issues that need to be improved.

**Suicide loss survivor is now defined in lines 133-135.**

2. Introduction: The authors need to elaborate more on the types of suicide and the suicide behaviors in the introduction for clear understanding of the topic, the risk factors, perception and implication to the affected individuals and communities. The authors should also elaborate more on the World Health Organization (WHO) and the International Association for Suicide Prevention formulated guidelines.

**We have expanded on the WHO and IASP guidelines in lines 55-67.**

3. Methods: The authors need to expand on the methodology. The authors should replace “Methods” with “Methodology”. In line 85, the authors state that “Deaths of all ages that occurred between 2019 and 2022….” what about those that were not reported or recorded? The authors should explain the study setting and the study population, the selection criteria, sampling procedure given that this is a sensitive topic. How were the “Suicide loss survivors” identified? And who are the “adult member” of the survivor? How was the pilot testing of the questionnaires conducted, and on how many participants and from where?

**We have replaced methods with methodology (line 86). Extensive details on the methods are now added (lines 104-126). The study sample of suicide deaths comes from the larger national study of all deaths of all ages. Suicide survivor and adult family member is now defined in lines 133-135. Pilot testing of the questionnaire is now added in lines 153-157.**

4. Results:

- The authors should check the numbers if they add up to the total number of the study participants as well as the 100% in the table 1.

**The numbers for each variable add upto 100% for male and female suicide deaths.**

- And a table for the total number and % suicide deaths for both male and female should be included in the table 1. The values in the tables indicate what?

**We did not include a column to show combined male and female data as the data for these two is quite different. It is important to bring about this gendered difference in the findings. Also, writing the findings and interpretation of data based on averaging two these two will not be meaningful. We would prefer to keep the table as is. The values in the table indicate “coverage of newspaper reporting of suicide death” as indicated in the column header.**

- The same should be done in table 2.

**Same as above.**

- Generally table 1 and 2 are not very clear and the text that follow them, and they need improvement.

**We have updated the language and also deleted some text as per the comments of Reviewer 2.**

- There is need to improve on the grammar in table 3.

**The sentences shown are translated as is from the verbatim. We have not attempted to correct the grammar to maintain authenticity of what was said by the respondents. We leave this to the Editor to decide if grammar update is needed.**

- Figure 1 and 2, need to be labeled and otherwise which is figure 1 and 2; and they need also to be improved on since they are not clear.

**Apologies for this oversight. These are now labelled and clarity is improved.**

5. Discussion: The authors need to improve on the discussion based on their findings, and they need to link their finding with previous studies and the implication of their finding. What were the limitations of the study?

**We are not aware of previous studies that have documented survivor perception of the media reporting of suicide death in settings such as ours. We have improved discussion by integrating case narratives (lines 318-347) and by detailing recommendations (lines 362-377). The limitations of the study are now indicated in lines 303-315.**

6. Conclusion: The authors need to separate the conclusion from the discussion. And the conclusions made do not seem to come from the authors’ findings i.e. results of the study.

**Thank you. We have re-worked the last paragraph (lines 366-377).**

7. References: There are some references that are incomplete such as the reference 1 and the authors need improve on the reference section.

**Thank you. We have now checked and updated.**

**Reviewer #5**

I believe it is an interesting and valuable report on an important matter. The statistical and overall quantitative analysis appear to be well conducted and reported. However, there is an important issue about the qualitative data, as it is not explained the analysis process in the methods.

**Thank you, we have improved the manuscript in this revision.**

For example, in the Table 3 there is a selection of quotes from the participants, but there is no information about how that selection was made. The thematic analysis should be explained in methods. This appears to me as a mixed methods paper and it should presented that way.

**We have now added these details in lines 163-166.**

In the Discussion, some statements lack a more formal or scientific language, for example when they refer to a "symbiotic" relationship between journalists and police force. That should be described in better terms.

**We have explained the relationship clearly now in lines 297-300.**

**Reviewer #6**

1. Introduction and Purpose: The study under review explores an under-researched area: how newspaper coverage of suicide deaths is perceived by suicide loss survivors in India. While global literature highlights the role of media in shaping public understanding of suicide, very little attention has been paid to how bereaved families experience such reporting. This research attempts to bridge that gap by examining both the extent of newspaper coverage and survivors’ support or opposition to it, with particular attention to socio-demographic factors.

**Thank you.**

2. Methodology: The researchers conducted semi-qualitative interviews with a key adult survivor from each of 155 suicide cases across three Indian states. The design is noteworthy for combining quantitative measures (e.g., proportions of cases reported in newspapers, statistical associations with socio-demographic variables) with qualitative insights (survivors’ stated reasons for support or opposition). A strength of this design lies in its relatively large and geographically diverse sample, which enhances generalizability compared to single-site or small-sample studies. However, the reliance on one survivor per case introduces a potential bias, as perceptions may differ within families. Moreover, the “semi-qualitative” approach is not fully detailed, leaving questions about the rigor of the qualitative analysis (e.g., coding methods, inter-rater reliability).

**Thank you. We have now replaced “semi-qualitative” with semi-structured interviews as describes what was done more appropriately. We have added details in lines 140-149. Also, we now refer to reliance on one respondent as a limitation in lines 313-315.**

3. Findings: The study reports that 39.4% of suicides were covered in newspapers, with slightly higher coverage of female suicides (43.2%) than male suicides (37.8%). Coverage was more likely when deaths were registered with the police. Survivors’ responses to such coverage varied:
   49.2% opposed it,
   31.1% supported it,
   19.7% declined to respond.

Support for coverage was linked to raising awareness about suicide, especially regarding farmer suicides and the broader implications of suicide. Interestingly, support for reporting male suicides rose with survivors’ education level, whereas female suicide reporting showed no clear socio-demographic patterns. Opposition was more common but often lacked articulated reasons.

**No response required.**

4. Strengths of the Study: Novel focus: Few studies consider survivors’ perceptions of suicide reporting, particularly in the Indian context, where stigma, cultural norms, and media practices intersect in complex ways.
   Empirical contribution: The study provides rare quantitative data on the prevalence of suicide reporting in newspapers, alongside survivors’ attitudes.

Policy relevance: Findings have clear implications for shaping media guidelines on sensitive suicide reporting.

**Thank you.**

5. Limitations

Analytical depth: The study’s qualitative findings are somewhat thin, as nearly half of survivors opposing coverage provided no reasons. More probing interviews or richer analysis could have deepened insights.

**Exploring how families experience media coverage of suicide and their perspectives on such reporting is a novel area of inquiry in India. Many bereaved individuals expressed opposition to media coverage of their loved one’s death, although they often could not provide specific reasons. Given the scarcity of opportunities for open discussion about suicide and its portrayal, these difficulties in articulating views are not unexpected. We have now indicated this in lines 326-331.**

Contextual gaps: The study does not analyze the content or tone of newspaper reports (sensational vs. factual), which likely influences survivors’ perceptions.

**This was beyond the scope of this work. We have identified this as a limitation in lines 310-311.**

Cultural considerations: While the study mentions farmer suicides, it does not fully engage with stigma, religion, or social honor, which are central to how suicide is perceived in India.

**Given the limited prior research on how families experience media reports of suicide deaths, the study was intentionally exploratory. We hope to undertake more in-depth work following these findings.**

Methodological ambiguity: The “semi-qualitative” approach lacks detail, limiting replicability and transparency.

**We have replaced semi-qualitative interviews with semi-structured interviews, as the latter reflects the methods more appropriately. We have also added details of how the interview was done in lines 135-140.**

Cross-sectional design: Survivors’ views may evolve over time, but the study captures only a single point in bereavement.

**We have added this as a limitation in lines 311-313.**

6. Implications: The study underscores the ambivalence of survivors toward suicide reporting: while some see it as an opportunity for awareness, others experience it as invasive or stigmatizing. This tension points to the need for:
   Culturally sensitive media guidelines tailored to Indian contexts.
   Training for journalists on responsible suicide reporting, balancing awareness-raising with survivor protection.
   Further research combining content analysis of media reports with longitudinal interviews of survivors to capture evolving perceptions.

**Thank you for these excellent suggestions. We have added these points in lines 358-377.**

7. Conclusion: This article makes a valuable contribution by foregrounding survivors’ voices in the discourse on media and suicide. It demonstrates that the impact of newspaper reporting is neither uniformly harmful nor beneficial but varies by socio-demography, education, and cultural context. While methodological limitations constrain the depth of its conclusions, the study opens an important research agenda on suicide, media, and bereavement in India.

**Thank you.**

Thank you for considering the revised version of our manuscript. If there more questions, we would be pleased to respond.

Best wishes

Rakhi Dandona
